# Evaluating a young-onset dementia service from two sides of the coin: staff and service user perspectives

**DOI:** 10.1186/s12913-020-5027-8

**Published:** 2020-03-06

**Authors:** Clarissa Giebel, Cassie Eastham, Jacqueline Cannon, John Wilson, Janet Wilson, Anna Pearson

**Affiliations:** 1 Institute of Population Health Sciences, University of Liverpool, 1st floor B Block Waterhouse Building, Liverpool L69 3GL, England, UK; 2 NIHR ARC NWC, Liverpool, UK; 3 North West Boroughs NHS Foundation Trust, Warrington, UK; 4 The Lewy Body Society, Wigan, UK

**Keywords:** Young onset dementia, Service evaluation, Service access, Focus groups, Post-diagnostic support

## Abstract

**Background:**

People with young-onset dementia (YOD) can often struggle getting the right treatment. This is because of their frequently different characteristics and needs compared to people with late-onset dementia. The aim of this project was to assess a memory service for its adaptation to the needs and wishes of people with YOD and their carers.

**Methods:**

This project evaluated a memory service in the North West of England by performing two focus groups with clinical staff and six semi-structured interviews with people with YOD and carers. The focus groups took place on site and lasted one hour each. People with YOD and their carers were identified via the memory clinics caseload and via the local Alzheimer’s Society charity organisation. Both focus groups and interviews were audio-recorded and transcribed, and data were analysed using thematic analysis. The public (a person living with YOD and his carer) were involved from the design stages of the project through to the analysis and dissemination.

**Results:**

Eleven members of staff with different clinical backgrounds participated in the focus groups and six interviews were held with people with YOD and their carers. Both indicated that whilst the diagnostic process is relatively well conducted at the service, the post-diagnostic service has many gaps. These include limited post-diagnostic support by the service, better enabling peer support, as well as providing meaningful activities, as some activities provided might be more suitable to older adults with dementia.

**Conclusions:**

Post-diagnostic services and support for people with YOD and their carers need to be improved. The next step will be to implement the findings from this service evaluation in practice and improve service satisfaction and relevance to people with YOD.

## Background

Dementia is mostly diagnosed above the age of 65 [2]. However, a minority group receive this diagnosis below this cut off age [25, 27] – in the UK there are estimated over 40,000 people living with young-onset dementia (YOD) alone, of an estimated total of 850,000 people with dementia [4.7% of all cases thus have YOD] [2]. YOD encompasses a range of subtypes, most commonly Alzheimer’s disease, followed by vascular dementia and frontotemporal lobar degeneration [25].

Whilst YOD affects a minority group of people with dementia, people with the diagnosis as well as their carers require specific support which can often vary from that provided to people with late-onset dementia. Similar to late-onset dementia [13], people with YOD experience difficulties in performing everyday activities such as preparing a hot meal or doing household tasks, and experience different levels of cognitive and behavioural symptoms, depending on the type of dementia [12, 17, 23]. As the term suggests, people with YOD are much younger and thus may be in employment or are caring for children or their own parents. By contrast, people with late-onset dementia are in most cases already retired and may only be caring for their partner, if at all. Thus, once a diagnosis of YOD is made, this can have several negative implications both financially as well as personally [5, 24], suggesting that people with YOD have different needs and require different types of post-diagnostic support [1].

It is not only the person with YOD is affected by the diagnosis, but family members who become their carers are also impacted upon. What characterises their experiences the most are the levels of stress and loss that carers often experience once their loved one has received a diagnosis. When interviewing young spouses of people with YOD, Lockeridge and Simpson [19] reported carers to engage in denying the situation as a coping mechanism, to report stigma, lack of control, and feelings of loss. Children also represent a large group of carers, and increasing evidence shows that adult child carers experience similar stresses to spousal carers [4, 15, 18]. This supports the need to involve the family in the post-diagnostic process and to provide family-oriented support.

Services not only need to be adapted to support the person with YOD post-diagnosis, but also their family carers. Research suggests that carers of people with YOD require more care support due to the higher levels of behavioural disturbances than in late-onset dementia [3]. However, growing evidence suggests that services are often maladapted to the needs and wishes of people living with the condition and their carers ([8, 9, 16, 20, 21]; 2018). The barriers to post-diagnostic services span from insufficient information [16] to not receiving support and help at the right time [10]. In particular, services often fail to provide sufficient support because of temporary and time-limited funding (for example by local councils) and thus often discontinue [20]. Whilst there is a growing literature on the shortcomings of services for people with YOD and their carers from the perspectives of service users, it appears that there is little to no evidence currently on staff perspectives on what should be adapted to accommodate and support service users better.

The aim of this exploratory service evaluation was to assess the provision and experiences of providing post-diagnostic support to people with YOD and their caregivers, from the perspectives of staff and service users. This service evaluation is the first step in highlighting potential shortcomings or elements of good practice, which in the second step of the process can be addressed via suitable staff training and implemented through implementation science.

## Methods

This service evaluation formed part of the National Institute of Health Research’s Collaboration for Leadership in Applied Health Research and Care North West Coast’s (NIHR CLAHRC NWC) Partner Priority Programme, now part of the new NIHR Applied Research Collaboration (ARC) NWC. The programme was set up to enable clinicians and partner organisations to evaluate topics of relevance to their organisation, in which co-production of research with frontline workers, academics, and members of the public was strongly embedded.

### Service description

Wigan Later Life and Memory Service (LLAMS) provides diagnostic and post-diagnostic support to people living with dementia, as well as supporting older people with complex functional mental health needs. The service has four teams: Assessment, Memory, Community Mental Health (CMHT), and Care Home Liaison. GPs are the most frequent source of referrals, but referrals are also accepted from other health and social care professionals, emergency services, family members or self-referral. After initial assessment and discussion with the consultant psychiatrist, cases of suspected dementia are transferred to the Memory team for further diagnostic investigations.

The Memory team is multidisciplinary and comprises of consultant psychiatrists, senior nurse practitioners, occupational therapists, counsellors, psychologists and support workers. Following a diagnosis, people with dementia and their families are offered counselling, post-diagnostic education groups and referral to the Dementia Advisers, who are provided by the local Alzheimer’s Society. Where appropriate, pharmacological management and non-pharmacological interventions, such as cognitive stimulation therapy are also provided. If the person has completed their post-diagnostic support, is stable on any prescribed medication and has no other complex needs, they will be discharged to the GP.

### Participants and recruitment

The Health Research Authority decision tool (HRA 2018) was used to determine that the project was a service evaluation and not research. As such, ethical opinion was not sought from a REC, however the service evaluation was registered with the Research and Development Department at North West Boroughs Healthcare NHS Foundation Trust. According to the UK Policy Framework for Health and Social Care Research, service evaluations are not considered research as such and therefore do not require ethical approval from an ethics committee.

People with dementia were eligible to participate in the project if they had a diagnosis of YOD (thereby having been diagnosed below the age of 65), and lived in the geographical area within the North West Coast region. Thus, we only recruited participants who were service users and had experiences of the service. Participants were living in the community, and people with YOD were excluded if they had acute mental health needs, such as severe depression, had a learning disability or lived in a long term care facility. Carers were included in the project if they were unpaid family carers or friends caring for the person with YOD on a regular basis. People with YOD and carers were identified either by being on the current memory clinic caseload or via the local Alzheimer’s Society and having completed a questionnaire stating their interest in the project. A participant information sheet was provided to people who expressed interest in taking part and opportunities to ask questions were provided throughout the recruitment and consent processes. Consideration was given to the issues of mental capacity and consent. Participants needed to have capacity to consent to taking part in the evaluation. Where the person with dementia did not have capacity to consent, the questionnaire or interview was conducted with a proxy such as a family member.

A convenience sample of clinical staff was recruited from Wigan Later Life and Memory Service (LLAMS). Staff members worked for either the memory team or CMHT and were required to have experience of being involved with the diagnosis or support of people living with young onset dementia. Potential participants were told about the focus groups in their team meeting and then provided with participant information.

### Materials and data collection

Focus groups with staff were guided by a semi-structured topic guide, addressing the suitability of the service to people with YOD and their carers. Both focus groups took place at the memory clinic and lasted approximately one hour, and were led by CE and CG.

Semi-structured interviews with people with YOD and carers took place in the participant’s home, and lasted approximately one hour. Interviews were led by CE who was trained in qualitative data collection and had experience in working with people with dementia and caregivers as part of her role as occupational therapist. Where people experienced difficulties answering a question and provided little detail, the interviewer provided prompts. Focus group and interview guides were developed for the purpose of this study (please see Supplementary File 1 and 2). Prior to conducting the focus groups and interviews, written informed consent was obtained. Both focus groups and interviews were audio recorded and subsequently transcribed, and the researcher made some field notes during the focus groups and interviews to guide subsequent analysis.

### Public involvement and co-production

This project has involved one person living with YOD and his spousal caregiver from the beginning. Both public advisers were recruited from the NHS Trust as having accessed the service in the past. For this project, both attended regular steering group meetings and provided feedback and input on project documents, such as information sheets. The steering group also comprised representatives from the local Council, the Alzheimer’s Society, Wigan Dementia Action Alliance, a volunteer Trust governor, and the NHS Trust R&D group, who were all actively involved in co-producing the project. Public advisers received a fee for attending meetings and for commenting on documents, and had their travel expenses reimbursed. Public advisers have also shaped the analysis and interpretation of the findings and have provided feedback on this paper.

### Data analysis

Data from the focus groups and the interviews were analysed using the principles of thematic analysis [7], and were coded by one of the research team members trained in qualitative analysis. Data were coded by hand, and no software was used.

## Results

Both staff focus groups were attended by a total of 11 members of staff (n_1_ = 8; n_2_ = 3), including support workers, occupational therapists, and community psychiatric nurses. In total, six interviews were held (one with a person with YOD, four with caregivers, and one with both). PwD were on average 61 years old and 50% were female. Caregivers were on average 55 years old and mostly female (80%). Caregivers included spouses, siblings, and adult children. Participants were interviewed in their own home.

Both focus groups and interviews provided insights into understanding how the service is currently managing in supporting people with YOD and how the service could be improved at three different stages of the diagnosis: Making the diagnosis; giving the diagnosis; living with the diagnosis.

### Making and giving the diagnosis

People with YOD and their carers felt that the diagnosis was conducted well without many difficulties, but some mentioned the length it took in order to receive a diagnosis. This is corroborated by staff also highlighting how considerate they are when making the diagnosis. Staff do not tend to rush the diagnosis of YOD, despite family members at times wishing for a quicker diagnosis process.

*“I think that because I was younger the GP was very sceptical because it was like, ‘have you got a family history?’. No. And it was sort of, it could be the menopause, but I knew specifically that the symptoms I had … were dementia symptoms. They kept saying to me ‘are you depressed?’ and I said, ‘I know I’m not depressed, you know. I just know that the things I do are not normal’”.*

Person with YOD 03

*“His wife wanted the diagnosis quickly, but because of the age you don’t want to just do that; give a diagnosis and it’s the wrong diagnosis. You want to make sure everything’s done before we do that, because as you say it affects everything doesn’t it?”*

Nurse, Memory Team (Focus Group 2)

### Living with the diagnosis

The small number of people with young onset dementia affects how LLAMS is able to provide education and activity groups for this population. The flow of people through the service limits the number who is open at any one time; as such, there are not enough people to justify running separate groups for them. Staff were concerned that by waiting for there to be enough people to run a group, people’s presentation would have deteriorated and education groups would no longer be appropriate.

*“And I think as well like from memory service point of view is that we don’t get, there’s they’ll come through drip by drip so you’ve not got a lot of people at that time to get together for doing a group and obviously the way we work we’re an early diagnostic treatment service it’s in and then run, it’s moving through so if you haven’t got that cohort of early onset then you can’t set up a group, certainly from our service because of that.”*

Occupational Therapist, Memory Team (Focus Group 2)

*“I think in an ideal world it would be nice to offer an educational group just for that group, however we might only have two or three in the service at any one time and our groups are only really beneficial for people in the early stages of the condition, so if we’re keeping somebody for a year in order to wait to have a viable number for groups, it may no longer be of use to them, things have moved on hugely in a year in for this population so offering individual groups unfortunately just isn’t a goer as far as I can see. I can’t see a way around that, other than offering 1:1, which we already do.”*

Occupational Therapist, Memory Team (Focus Group 1)

The support and services that could be offered with LLAMS was also affected by the perception of being ‘too young to have dementia’. An important aspect of the memory service is the provision of post-diagnostic education groups for people with dementia and their carers. Younger people could seem out of place to other group members or could also feel that they did not fit within these groups. In particular, staff identified that seeing older people who may have more advanced symptoms could be frightening for younger people. Although the content of the group sessions can be tailored to meet the needs of the cohort, staff recognised that the value of these groups is greater than simply the information that is given.

*“I can’t see him and her in the same group … I asked what age they were and they said anything from something to something, and I thought ‘well is the majority going to be like old people?’ I’m not saying I’m not an old person, but you know what I’m saying? And I just thought, ooh no it’s not for us.”*

Family carer 02

*“It’s really difficult because they don’t want to be seen in a walking group with older people that are maybe walking a lot slower, maybe have more physical health problems, more mobility problems but there isn’t a great deal out there tailor made for that 1:1 support for someone with young onset”*

Nurse, CMHT (Focus Group 2)

*“I suppose some of the things that are said in those groups, maybe people who are mild to moderate it’s going to be scary as well. So it’s got to be kind of in a different environment really for those younger people.”*

Nurse, Memory Team (Focus Group 2)

Similarly, although there is a wide range of dementia-friendly activities available in Wigan, staff felt that younger people did not fit in at these. Finding appropriate placements for day care, long term care and supported accommodation was also challenging because younger people did not fit in at these services.

*“Another area that’s … been quite distressing is when people have needed care home support. There is absolutely no care home availability for a younger age group and they’re put with people in their 80s and 90s and it sticks out like a sore thumb, and they are, however impaired they are, very much are aware that they are not in the right setting for them. There just is no availability sadly for that population in terms of care homes.”*

Occupational Therapist, Memory Team (Focus Group 1)

*“Even though for years I’d worked with psychogeriatrics, I think the fact that when you’re facing a room with people who are maybe further along than you, although I know what’s coming, I don’t want to. I didn’t want to be in that group.”*

Person with YOD 05

## Discussion

This project evaluated a local memory service for its suitability to and service provision for people with YOD and their caregivers. Both members of staff and people with YOD and caregivers were primarily content with the service provided during the process of diagnosis, but highlighted several limitations to the provision of post-diagnostic support.

Once people with YOD are diagnosed, they receive access to a 6-week post-diagnostic education group which is designed for anyone with dementia, including late-onset dementia. Caregivers are also invited to attend. Due to the nature of the group and by opening up the group to people with young and late-onset dementia, the smaller proportion of people diagnosed with YOD often felt out of place and would have preferred more tailored support. This is because people with YOD have often specific needs, as evidenced in this project and in previous research [6, 11]. For example, people with YOD are often still in employment and struggle thus with finances or keeping hold of their job. As outlined in a recent report on the current national and international state of YOD specialist care, the UK is in many geographical areas lacking behind providing specialist YOD care [8]. In the Netherlands for example, there are age-specific units that provide more targeted support to people living with YOD, by for example providing more age-appropriate active activities [8]. It may be important for the service to engage more closely with third sector organisations providing activities, and work collaboratively in designing activities requested by people with YOD.

As staff outlined in the focus groups, often there are very few cases of YOD that go through the service simultaneously. Therefore, setting up a YOD-specific post-diagnostic support group can be difficult to manage. If the service were to wait until sufficient people with YOD have received a diagnosis however, the support may come too late to some people. This may be the result of the size of the service’s population. In comparison, a memory service serving a larger (urban) population may be better equipped at providing specific post-diagnostic support for people with YOD due to a greater number of cases going through the services at any given time.

Whilst all groups, including staff and people with YOD and their carers, highlighted this as a barrier, they also expressed concerns about the time-limited provision of post-diagnostic support in the first place. The Wigan LLAMS provides a 6-week standard post-diagnostic education group for people with late- and early-onset dementia. This includes providing information, signposting and sharing experiences with peers. However, not every person with a diagnosis wants to attend a support group and meet other peers. Indeed, men living with dementia can be found resisting accessing social support groups or day care services, as opposed to women [14]. In the interviews, service users have expressed a wish to have continued support to deal with and adapt to the diagnosis. Whilst the 6-week programme was considered useful, there were more long-term support needs that service users felt not addressed and supported with from the service. Similar needs were identified in a metropolitan memory clinic service evaluation involving people with any type of dementia and their caregivers [26]. A 6-week education programme is likely to provide too little support because once a diagnosis is made and the education group is finished, people with YOD are referred back to the GP, who is not providing any specialist services. One solution might be for people with YOD to be provided with a linked support worker in their post-diagnostic journey, as recommended in recent NICE guidelines on management and support of people with dementia and their caregivers (2018) [22].

Involving people with lived experience of dementia as well as the local council and third sector organisations has greatly benefitted this service evaluation. Public advisers (1 person with YOD and his spousal caregiver) and partners have coproduced this project from the design process, through to the analysis and write up. By discussing the progress of the research and involving these parties throughout all stages of the evaluation, changes have been made. Particularly, they made important contributions to the interpretation of the findings, and what the next steps of implementing changes could be.

### Limitations

This is a service evaluation of a local memory service within the North West Coast area of England, so that findings from this project are not representative on a national level. Participants could therefore only be recruited from the Wigan LLAMS, which can limit the number of participants. Thus, data might not reach data saturation, although themes emerged by several participants. Moreover, we conducted both interviews and focus groups, thereby gathering a varied number of perspectives on the topic, with findings supporting and extending previous evidence showing that services, especially post-diagnostic services, for people with YOD are often not suitable and require adaptation to fit the needs and wishes of this population [8, 16]. It is important to highlight though that the findings are based on a small number of exploratory focus groups and interviews, so that conclusions drawn from the data need to be considered in the light of the small representativeness of participants. Only one researcher (CE) analysed the transcripts, which were then subsequently discussed with another research team member (CG) to generate themes.

## Conclusions

Services for people with YOD and their caregivers need to be better adapted to the service users’ post-diagnostic support needs. Most importantly, both staff and service users and their caregivers expressed a need for a continuous support link after being referred back to the GP. Future work should look at implementing such a support worker or similar professional role in the local service or in other similar services, and services are recommended to work closely together with the local providers to design and provide activities suited for people with YOD and their caregivers. It is important that an implementation of such a link contact is guided and coproduced again with service users, caregivers, clinical staff as well as third sector organisations involved in supporting people with YOD.

## Supplementary information

**Additional file 1.**

**Additional file 2.**

## Data Availability

This is a qualitative study. Anonymous qualitative data supporting the conclusions of this article can be accessed by contacting the lead author.

## Abbreviations

CMHT: Community Mental Health Team
LLAMS: Later Life and Memory Service
NIHR CLAHRC NWC: National Institute of Health Research’s Collaboration for Leadership in Applied Health Research and Care North West Coast’s
YOD: Young onset dementia
