# Supplementary material for: Evaluating a young-onset dementia service from two sides of the coin: staff and service user perspectives
Source: BMC Health Serv Res. 2020 Mar 6;20:187. doi: 10.1186/s12913-020-5027-8 (PMC7060597; doi:10.1186/s12913-020-5027-8)
Supplement: Supplementary file 1 — Additional file 1. [file 12913_2020_5027_MOESM1_ESM.docx]

Introduction

**Opening questions**

We’d like you to think about the times that you’ve worked with people who are under-65.

- Would anyone like to talk about their experiences?
- What might be different when working with someone who is younger?
  - Finances
  - Work
  - Caring responsibilities
  - Family relationships
  - Physical health

**Identifying needs**

This is taken from the Young Dementia Networks recommendations for assessment and support should be provided to younger people. Have a read and see what you think.

- What are your thoughts on those recommendations?
  - Life etc before diagnosis and afterwards
  - Coping strategies
  - Circumstances – work, finances, family
  - Family circumstances
- As a service, what do you do to identify people’s needs?
  - How does the service identify people’s individual needs?
  - Are there any challenges to doing this?
- Is there anything the service doesn’t do or could do better?

**Supporting people**

Again, these recommendations are from the Young Dementia Network.

- What are your thoughts?
- As a service, what do you do to support people’s needs?
  - Which other services do you refer people to?
  - Are there any challenges to doing this?
- Is there anything the service doesn’t do or could do better

**Age appropriate services**

A lot of work around young dementia services says that they should be “age appropriate”.

- What do you think is meant by “age appropriate”?
  - Does LLAMS provide services that are age appropriate?
  - Is there anything that the service could to improve this?
- Are there any barriers to younger people accessing LLAMS?

**Staff training**

- Has anyone had any specialist training about young onset dementia?
  - Good/bad
  - Content
  - Useful
- Ask people to self-rate their knowledge and skills privately (cards in envelopes).
- Is there anything you would like to have more training or information about?

**Summary**

- Is there anything that LLAMS does well when working with younger people?
- Is there anything that needs to be improved?
- Do you have any other thoughts or views you would like to share?
