# Supplementary material for: Evaluating a young-onset dementia service from two sides of the coin: staff and service user perspectives
Source: BMC Health Serv Res. 2020 Mar 6;20:187. doi: 10.1186/s12913-020-5027-8 (PMC7060597; doi:10.1186/s12913-020-5027-8)
Supplement: Supplementary file 2 — Additional file 2. [file 12913_2020_5027_MOESM2_ESM.docx]

**Interview Guide**

Demographic details.

Ages. Relationship. Carer?

Diagnosis. When made – year/age?

Going back to the beginning of your experiences, what happened at the start when you noticed some changes?

GP. Specialist services. Symptoms. What did you think was happening? Willing to seek diagnosis?

What was your situation like at that time?

Work. Family. Physical health. Finances.

What happened when you started to look for a diagnosis?

Any difficulties? Anything easy?

How well did the memory service identify what you needed?

Good/bad.

What did the memory service do to support you with your needs?

Good/bad. Carer/PWD.

Education groups. Dementia advisors. Groups. Activities. Community.

What other services have you used?

OOA.

Anything particularly useful?

Is there anything you would have like to have done or had support with that wasn’t available?

Have you been able to meet any people in a similar situation?

Was the service appropriate for your age?

Was there anything about your experience that you think was different because of age?

Different than those who are older?

What would improve services for younger people?

Magic wand?

Is there anything else you want to tell us about?

Is there anything we could do to improve this experience?
